# The Value of Ultrasound for Detecting and Following Subclinical Interstitial Lung Disease in Systemic Sclerosis

**DOI:** 10.3390/tomography10040041

**Published:** 2024-04-03

**Authors:** Marwin Gutierrez, Chiara Bertolazzi, Edgar Zozoaga-Velazquez, Denise Clavijo-Cornejo

**Affiliations:** 1 Center of Excellence in Rheumatic and Musculoskeletal Disorders, Mexico City 03530, Mexico; chiara.bertolazzi@gmail.com; 2 Division of Rheumatology, Instituto Nacional de Rehabilitacion Luis Guillermo Ibarra Ibarra, Mexico City 03530, Mexico; dence.cc@gmail.com; 3 Pneumology Department, Unidad Medica de Alta Especialidad, 1 Bajio, Leon 37680, Mexico; zozoaga@gmail.com

**Keywords:** pulmonary ultrasound, systemic sclerosis, subclinical, interstitial lung disease

## Abstract

Background: Interstitial lung disease (ILD) is a complication in patients with systemic sclerosis (SSc). Accurate strategies to identify its presence in early phases are essential. We conducted the study aiming to determine the validity of ultrasound (US) in detecting subclinical ILD in SSc, and to ascertain its potential in determining the disease progression. Methods: 133 patients without respiratory symptoms and 133 healthy controls were included. Borg scale, Rodnan skin score (RSS), auscultation, chest radiographs, and respiratory function tests (RFT) were performed. A rheumatologist performed the lung US. High-resolution CT (HRCT) was also performed. The patients were followed every 12 weeks for 48 weeks. Results: A total of 79 of 133 patients (59.4%) showed US signs of ILD in contrast to healthy controls (4.8%) (*p* = 0.0001). Anti-centromere antibodies (*p* = 0.005) and RSS (*p* = 0.004) showed an association with ILD. A positive correlation was demonstrated between the US and HRCT findings (*p* = 0.001). The sensitivity and specificity of US in detecting ILD were 91.2% and 88.6%, respectively. In the follow-up, a total of 30 patients out of 79 (37.9%) who demonstrated US signs of ILD at baseline, showed changes in the ILD score by US. Conclusions: US showed a high prevalence of subclinical ILD in SSc patients. It proved to be a valid, reliable, and feasible tool to detect ILD in SSc and to monitor disease progression.

## 1. Introduction

Interstitial lung disease (ILD) is a frequent complication in systemic sclerosis (SSc) patients and despite recent advances in treatment, it is still the major cause of death of these patients [1,2]. ILD usually may be established during the first 4 years of the SSc being frequently subclinical [3,4]. Thus, particular attention in detecting this complication early is a crucial need in the clinical setting, because it may lead to a poor prognosis and quality of life. So, an accurate strategy is desirable to detect ILD in its early (or preclinical) stages.

Beyond the clinical history, currently, to assess the presence of SSc-ILD, there are different tools including respiratory functional tests (RFTs) and imaging methods such as X-ray and high-resolution computer tomography (HRCT).

As previously mentioned the clinical manifestations might be ambiguous or absent in the initial stages of SSc-ILD [5]. Moreover, RFTs can show unspecific or normal findings despite an established ILD [6,7]. In this context, imaging can play a key role in detecting the earliest SSc-ILD.

In the past, chest radiography was the most frequent imaging method adopted to assess ILD, but currently, its application has dramatically reduced due to the low sensitivity in the initial stages of ILD.

HRCT is the most common imaging technique used in the assessment of ILD. It is the gold-standard imaging method reference for the diagnosis/prognosis, the quantification of severity, pattern analysis, and therapy monitoring of ILD [8,9]. Furthermore, it has shown the ability to detect both early pulmonary changes and subclinical lung involvement [8]. Despite these qualities, its routine use is very limited due to high costs, scarce availability in non-tertiary health centers, long waiting times in some realities, and ionizing radiation. This latest aspect is of relevance since the issue of radiation exposure has recently been raised [10,11]. In this context, pulmonary ultrasound (US) has emerged as a potential tool for the assessment of ILD in patients with SSc [12,13,14,15,16]. In fact, in recent years, pulmonary US has been demonstrated to be reliable, feasible, and valid for the assessment of SSc-ILD [17,18,19].

Despite the growing body of evidence supporting its utility in ILD, there are no solid data regarding its potential role in both detecting ILD in subclinical stages and monitoring the evolution of ILD in these SSc patients. Taking into account this gap of knowledge, we decided to investigate the validity of pulmonary US in detecting subclinical ILD in SSc and to determine its potential role in monitoring the ILD progression.

## 2. Material and Methods

### 2.1. Patients

We included consecutive patients with a diagnosis of SSc according to the 2013 ACR/EULAR classification criteria for SSc [20], attending the outpatient and inpatient clinics of the centers involved in the study.

The inclusion criteria included age > 18 years, non-smokers, and patients with a diagnosis of SSc without previous or current respiratory symptoms (including dyspnea or cough), or previous imaging tests reporting ILD. Patients with a previous diagnosis of ILD or pulmonary diseases such as chronic obstructive pulmonary disease and pulmonary edema were excluded mainly to avoid overlap of lung findings.

### 2.2. Study Design

All patients underwent a complete clinical evaluation by an expert rheumatologist in order to confirm the absence of respiratory symptoms. Particular attention was paid to the detection of fine basilar dry inspiratory crackles or “velcro sound” at the lung auscultation. Rodnan skin score (RSS) and Borg dyspnea scale (Borg score) were also additionally performed in all patients.

Chest X-ray and RFTs were performed on the same day in all patients. Successively, pulmonary US was performed by a rheumatologist expert in lung examination (with more than 12 years of experience in US) who was blinded to clinical and laboratory assessment.

To determine the concurrent validity, HRCT was performed within the 7 days after pulmonary US assessment by an operator who was blinded to clinical, RFT, and pulmonary US findings. Finally, serologic tests (anti-centromere, anti-Scl70) were obtained from all patients.

In order to evaluate the inter-observer reliability, a second rheumatologist with 1 year of US experience, who received previously a 1-month dedicated and intensive training in lung US, scanned the half of patients. A healthy group, sex and age-matched, was included as a control group.

The patients were followed every 12 weeks for 48 weeks performing pulmonary US, RFT, and Borg scale at each visit.

### 2.3. US Assessment

US examinations were assessed using a GE Versana Premier machine (GE Medical Systems, Inc. Boston, MA, USA) provided by a 5–13 MHz linear transducer or 3–5 MHz convex transducer. The patient positions for the US examination and the scanning technique were those previously described [16]. Briefly, for the anterior chest, the 2nd lung intercostal spaces (LIS) along the para-sternal lines, the 4th LIS along the mid-clavear, the anterior axillary, and the mild-axillary lines were assessed. For the posterior chest, the 8th LIS along the paravertebral, the sub-scapular, and the posterior axillary lines were assessed. Patient positions were supine or near-supine for the anterior chest scanning, while in a sitting position for the posterior chest scanning.

### 2.4. US Interpretation

**Figure 1:**
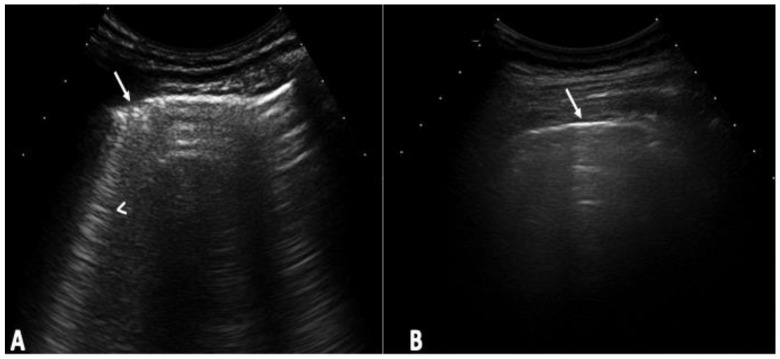
US scans of the lung surface in transversal scan with the probe positioned in the intercostal space: (**A**) Pulmonary US of patient with ILD. Note the B-line (arrowhead) as a hyperechoic narrow-based reverberation, spreading like a laser ray up to the edge of the screen. Note the pleura is irregular in this site (arrow). (**B**) US examination of healthy interlobular septa at lung surface level. Note the pleura is a linear and regular hyperechoic band (arrow). There is no presence of B-lines.

The US elementary lesions evaluated were the US B-lines (Figure 1) [21,22]. The US B-line total sum of all LIS was recorded and classified according to the following semi-quantitative US scale [0 = normal (≤5 B-lines); 1 = slight (≥6 and ≤15 B-lines); 2 = moderate, (≤16 and ≥30 B-lines); 3 = severe (≥30 B-lines)] [16]. The simplified score was obtained by a simple post hoc analysis resulting from a comprehensive US assessment. The semi-quantitative score including the cut-off was chosen because it demonstrated both a higher prevalence of US B-lines in the comprehensive assessment and easy accessibility by US.

### 2.5. HRCT Assessment

HRCT examination was performed using a CT 64 E Light-Speed VCT power scanner (BC Technical, Philadelphia, PA, USA) with a rotation tube with a scanning time of 0.65 s. Pulmonary involvement was evaluated by lung segments according to the Warrick score [23]. The severity of the disease was obtained by adding single-point values. The extension of the pulmonary involvement was obtained by counting the number of bronchopulmonary segments involved for each abnormality: one to three segments scored as 1; four to nine segments scored as 2; more than nine segments scored as 3. The total HRCT scores of severity and extension were calculated between the range from 0 to 30, as described previously [18]. The following semi-quantitative scoring was adopted to correlate accurately the US with HRCT findings: [0 = normal (0 points); 1 = mild (<8 points); 2 = moderate (from 8 to 15 points) and 3 = marked (>15 points)].

### 2.6. Statistical Analysis

Statistical analysis was performed using Stata v13.0 (StataCorp., Collage Station, TX, USA). Standard descriptive results were expressed both as means ± standard deviations (SDs) and medians. Categorical data were expressed as proportions. For the multivariate analysis (to determine associations between variables and US findings) a binary logistic regression was conducted with chi-square interpretation and momio reason. Spearman rank correlation coefficient (rho coefficient) was used for the correlation between the US and HRCT.

Accuracy, including sensitivity, specificity, and predictive values of X-ray, pulmonary US, pulmonary auscultation, and RFT, was measured by the area under the ROC curve.

ROC curves were created by plotting the true-positive proportion versus the false-positive proportion (sensitivity versus specificity respectively). The area under the ROC curve (AUC) was employed to quantify the discriminative accuracy of US. AUC from 0.50 to 0.70 represents poor accuracy, those from 0.70 and 0.90 are useful for some purposes, whereas higher values represent high accuracy.

To detect differences between US, RFT, and Borg score in the longitudinal assessment, Cochran’s Q test was used. For the inter-observer reliability of US findings, a weighted kappa statistic was adopted [24]. The feasibility was calculated according to the time spent during each US examination by the independent samples *t*-test (*p* values less than 0.005 were considered statistically significant).

## 3. Results

One hundred and fifty-four SSc patients were recruited to be included in the study. From those, 21 patients were excluded due to the presence of at least one exclusion criterion. The study was conducted definitively on 133 SSc patients and 133 healthy sex- and age-matched controls.

### 3.1. Baseline Assessment

A total of 1.862 LIS were globally scanned. Demographic and clinical data of the study population are reported in Table 1.

**Figure 2:**
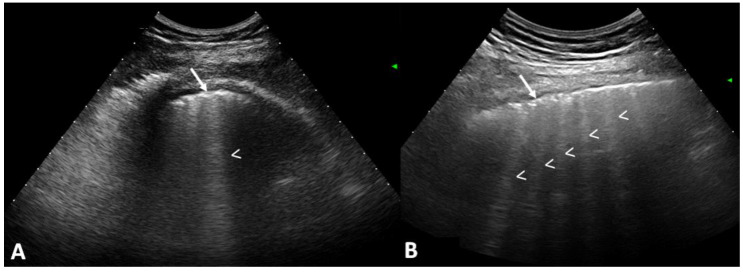
US scans of the ILD patients. The probe is in transversal scan in between the intercostal space. US examinations showing different scores of ILD involvement: (**A**) Mild. There is only a single B-line (arrowhead). (**B**) Moderate. There are six B-lines (arrowheads). Both cases present irregularity of the pleura (arrows).

A total of 79 out of 133 patients (59.4%) showed US signs of ILD with respect to the healthy controls (4.8%) (*p* = 0.0001). Figure 2 shows the US B-lines of SSc patients.

RSS and anti-centromere antibodies were the variables that showed association with pulmonary US-ILD (*p* = 0.003 and *p* = 0.005, respectively), whereas no association was found with gender, age, disease duration, chest radiography, and RFT findings (Table 2).

US B-lines showed a positive correlation with the HRCT Warrick score (rho = 0.802; *p* = 0.0001). A 90.2% concordance between the two imaging methods was additionally shown in the overall population, with a sensitivity and specificity of US with respect to HRCT of 91.2% and 88.6%, respectively. The 13 discordant cases were related to false positives in US (6 cases) and false negatives in 7 cases.

Sensitivity and specificity for the chest X-ray, pulmonary auscultation, and RFT in the assessment of ILD were 2.5% and 98.1%, 8.7% and 98.1%, 27.5% and 77.3%, respectively. Table 3 shows more details regarding the sensitivity, specificity, predictive value, and area under the ROC (AUC-ROC) curve. The AUC-ROC analysis confirmed the analytical relationship between the pulmonary US B-lines and the presence of ILD at HRCT.

The global kappa values for the inter-observer reliability of pulmonary US semi-quantitative score showed a good agreement between the two investigators (overall kappa = 0.72).

The mean time spent to perform the pulmonary US assessment was 8.6 min (±SD 1.4, range 6 to 12 min).

### 3.2. Longitudinal Assessment

All 133 SSc patients started the follow-up; however, 12 patients did not complete the 1-year follow-up (4 patients abandoned the study within the 3 months, 5 patients within the 6 months, 1 patient within the 9 months, and 2 patients within the 12 months). In total, 121 SSc patients completed the 1-year follow-up in the same hospital. 

A total of 30 out of 79 patients (37.97%) that showed US signs of ILD (B-lines) at baseline assessment demonstrated a progression in severity of ILD according to the US semi-quantitative score. Supplementary Table S1 shows the progression in terms of the number of B-lines from the baseline assessment to the 12-month follow-up.

**Figure 3:**
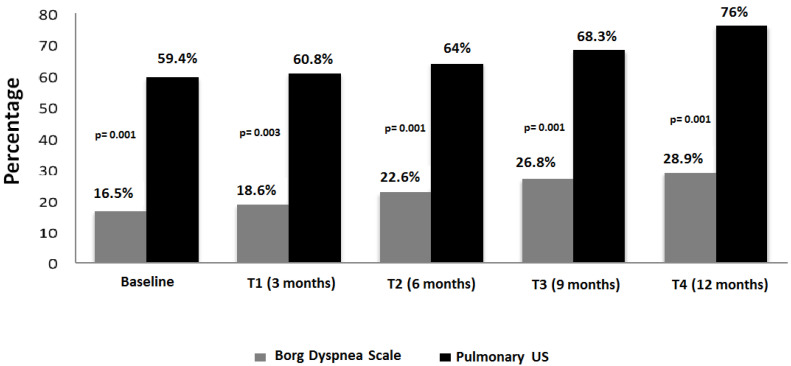
Progression of Borg Dyspnea scale and US findings.

Only 9 of those 30 patients (30%) developed respiratory symptoms during the follow-up. The elapsed time, in which the progression of ILD or clinical conditions was documented, was around 6 and 9 months of follow-up (Supplementary Table S2). The progression of both US and Borg dyspnea scale changes is illustrated in Figure 3.

## 4. Discussion

ILD is a leading cause of mortality in patients with SSc [1,2,3,4,5], so its early diagnosis is mandatory and represents one of the primary goals in order to improve the prognosis of the disease.

Although HRCT is, and will remain, the current gold-standard imaging method for the assessment of ILD in SSc patients, including diagnosis and prognosis purposes [25,26], pulmonary US provides interesting and solid data regarding its potential to assess SSc-ILD [27,28,29,30]. These researchers have provided important information regarding the correlation between pulmonary US and HRCT findings in SSc patients with established ILD [16,31,32,33]. Interesting data regarding the good performance of US in terms of reliability and feasibility of SSc-ILD were also recently documented [15,33,34,35,36,37]. These data open up an interesting research opportunity to consolidate the utility of pulmonary US as a biomarker of SSc-ILD.

Despite the emerging literature, there are no studies aimed to explore the diagnostic value of pulmonary US in the subclinical (or preclinical) stages of ILD and its potential role in monitoring ILD progression in SSc patients. 

Our results showed a high prevalence of subclinical ILD detected by pulmonary US (59.4%) in SSc patients despite the Borg score and RFT resulting in normal, or without, significant restrictive abnormalities (Table 1). We can interpret and confirm that ILD may be asymptomatic for several years before the symptoms appear [25,26]. However, it is not surprising since in autopsy studies, previously performed, 90% of patients showed ILD by HRCT [38,39], whereas, surprisingly, only 40–55% showed changes in RFT [40]. Our results also confirm the good sensitivity and specificity of pulmonary US in assessing ILD with respect to HRCT, whereas radiographs, pulmonary auscultation, and RFT showed poor sensitivity and specificity for ILD in preclinical stages.

Interesting data have also emerged from the follow-up of patients. A total of 30 (55%) out of 79 SSc patients who showed US signs of ILD at baseline showed a progression in their US score of ILD. However, only nine of those patients developed dyspnea. Although this number is very low, some considerations can be formulated: (a) pulmonary US can detect the progression of ILD despite the clinical scale for measuring dyspnea remaining unchanged, and (b) there is no relationship between the changes in the severity of ILD by the number of B-lines and the start of symptoms. Several patients changed their ILD status from mild to moderate, but not all developed respiratory symptoms (Supplementary Table S2).

From a further analysis of our results, additional considerations can be made. Firstly, the terms subclinical and/or preclinical could still be confusing in the routine nomenclature of the clinicians to describe interstitial lung abnormalities (ILA). However, it is important to underline that a recent expert position paper recommended that incidentally detected ILA in SSc patients should be classified as “preclinical or subclinical ILD” due to the presence of a risk factor for progressive disease [41].

Second, a chest radiograph confirms its scarce utility for the assessment of early phases of ILD as well as pulmonary auscultation, and RFT may fail in detecting early ILD. Third, US offers many advantages for ILD assessment. It is a widely available tool, inexpensive, and largely accepted by the patient. Moreover, portable machines can be sufficient for a detailed ILD assessment, as demonstrated previously [36] Fourth, pulmonary US cannot replace the meaningful information obtained with HRCT for the final diagnosis of ILD or for a complete evaluation of lung involvement; to the contrary, US could be considered as a complementary tool to implement in the very early stages of SSc (as a screening tool) in order to identify patients with the potential risk of developing SSc-ILD or to move on HRCT to determine the phenotype and prognosis evolution. However, to date, there is no evidence to recommend screening for early or subclinical SSc-ILD with HRCT [42].

The ability of US to identify early signs of ILD may position its routine use as essential in the management of patients who require serial examinations. Moreover, minimizing radiation exposure is important in SSc because of the observed puzzling relation between SSc and breast cancer, which usually appears on average 20 years after SSc onset [43,44,45].

According to our opinion and experience, in the near future, pulmonary US might take part in the algorithm of ILD diagnosis together with other examinations such as RFTs and HRCT.

We are aware that our study presents potential limitations. First, the low number of enrolled patients does not permit an accurate evaluation in terms of sensitivity and specificity, which could more strongly support these data. Second, an additional DLco, which is the most important functional test demonstrating a better correlation with the extent of lung fibrosis, was not performed [25]. It was omitted mainly because our institution requires additional costs in charging the patients. Third, an HRCT assessment was not performed during the longitudinal phase of the study, which limits more solid support for the usefulness of pulmonary US in monitoring the progression of ILD. HRCT was also not performed due to the costs and ethics aspects since it is difficult to justify a sequential HRCT assessment of SSc patients in the absence of respiratory symptoms. Despite this, we used RFTs and the Borg scale during the follow-up, which are tests largely used in real life to identify initial signs of ILD in SSc patients. Additionally, we believe that this is the first attempt to propose US for the follow-up. We currently are conducting an ancillary study including US and HRCT in the follow-up, which will give important information on the value of US for the monitoring of evolution and/or responsiveness. Fourth, the 12 months of longitudinal follow-up provide a limited time to support the role of US in monitoring the progression of ILD. However, although a minimal quota of patients developed respiratory symptoms (dyspnea), most of them (55%) showed an ILD progression by US during the 12 months, in the absence of symptoms. This aspect is of great interest in order to reflect on the clinical and US disparity, which seems independent of the evolution time. Ongoing follow-up of our cohort of patients will better elucidate this aspect. Fifth, the additional causes of dyspnea of those patients who developed it during the follow-up have not been accurately investigated in order to determine if the symptoms were closely related to the new-onset ILD. Sixth, only B-lines were considered as a US elementary lesion of ILD. B-lines are not specific for ILD as they can also be present in other heart and pulmonary pathologies. So, in this way, the HRCT will always be the gold standard for the characterization and quantification of ILD. Recently, pleural irregularity has been proposed as a new US lesion of ILD [46]. However, its clinical implication has not yet been consolidated. Seventh, our cohort seems inhomogeneous, considering the severity of the SSc. Finally, a comparison with a control group with other diseases involving the pulmonary interstitium would have added important information but was not considered. However, this has been addressed in previous papers, which showed data similar to our findings [16,36,37,38,39].

## 5. Conclusions

US is a valid, reliable, and feasible tool to detect very early (preclinical or subclinical stages) ILD and to follow its progression in patients with SSc. Despite a great deal of work that remains to be undertaken (construct validity, reliability, and sensitivity to change studies, in order to validate lung US as an outcome-measurement instrument in ILD-SSc), our preliminary results represent a milestone toward possible implementation, in the near future, of pulmonary US as an innocuous screening tool for the very early diagnosis of ILD in SSc and for monitoring its progression.

## Figures and Tables

**Table 1:** Demographics and characteristics of the study population.

Variable	Mean ± SD or (%)
Sex	
Male	13 (9.77)
Female	120 (90.23)
Age (years)	51.2 ± 10.2
Disease duration (years)	4.09 ± 2.3
Type of SSc	
Limited	57 (42.86)
Diffuse	76 (57.14)
Current treatment	
None	51 (38.35)
Methotrexate	35 (26.32)
Mycophenolatemofetil	40 (30.08)
Sildenafil	0 (0.00)
Azathioprine	2 (1.50)
Bosentan	2 (1.50)
Cyclosporine	1 (0.75)
Cyclophosphamide	2 (1.50)
Raynaud’s phenomenon	
Yes	115 (86.47)
No	18 (13.53)
Rodnan skin score	10.9 ± 7.9
Pulmonary auscultation	
Positive	0
Negative	133 (100)
Borg Dyspnea Scale	
0	130 (97.74)
0.5	3 (2.26)
Anti-topoisomerase (anti-Scl-70)	
Positive	123 (92.48)
Negative	10 (7.52)
Anti-centromere	
Positive	74 (55.64)
Negative	59 (44.36)
Chest X-ray	
Positive	3 (2.26)
Negative	130 (97.74)
Pulmonary US (semi-quantitative scale)	
0	54 (40.6)
1	51 (38.35)
2	28 (21.05)
3	0 (0)
HRTC (semi-quantitative scoring)	
0	53 (39.85)
1	58 (43.61)
2	19 (14.29)
3	3 (2.26)
FEV 1% predicted	93 ± 24.5
FVC% predicted	96.9 ± 26.2

HRTC = High-resolution computer tomography; FEV 1 = Forced Expiratory Volume in 1 s parameter; FVC = Forced Vital Capacity.

**Table 2:** Association between US signs of ILD and variables: summary of the results of binary logistic regression.

Variable	OR (CI 95%)	*p*
Sex	2.46 (0.64–9.41)	0.178
Age (years)	1.03 (0.99–1.07)	0.107
Anti-topoisomerase (anti-Scl-70)	2.34 (0.63–8.74)	0.205
Anti-centromere	2.80 (1.37–5.72)	0.005
Disease duration (years)	1.14 (0.98–1.34)	0.087
Raynaud’s phenomenon	0.70 (0.24–1.99)	0.501
Rodnan skin score	1.07 (1.02–1.13)	0.003
Borg Dyspnea scale (Borg score)	77.68 (3.86–1562.08)	0.086
Chest X-ray	1.37 (0.12–15.57)	0.796
RFT	0.98 (0.96–1.01)	0.144

RFT = respiratory functional tests.

**Table 3:** Sensitivity, specificity, predictive positive, and negative value and area under the ROC curve.

	Sensitivity	Specificity	PPV	NPV	AUC	95%IC
HRCT						
Chest X-ray	2.5	98.1	66.6	40	0.503	0.477–0.528
Pulmonary auscultation	8.7	98.1	87.5	41.6	0.503	0.498–0.570
RFT	27.5	77.3	64.7	41.4	0.524	0.449–0.599
Pulmonary US	91.2	88.6	92.4	87	0.899	0.846–0.952

HRCT = high-resolution computer tomography; PPV = positive predictive value; NPV = negative predictive value; AUC = area under the ROC curve; 95%IC = 95% confidence interval; RFT = respiratory functional test; US = ultrasound.

## Data Availability

Data are contained within the article and Supplementary Materials.

## Supplementary Materials

The following supporting information can be downloaded at: https://www.mdpi.com/article/10.3390/tomography10040041/s1, Table S1: Changes in number of the US B-lines at baseline and at the 12 months of follow-up; Table S2: Borg Dyspnoea scale and pulmonary US progression during 1-year of follow-up.
