# Supplementary material for: The Value of Ultrasound for Detecting and Following Subclinical Interstitial Lung Disease in Systemic Sclerosis
Source: Tomography. 2024 Apr 3;10(4):521–32. doi: 10.3390/tomography10040041 (PMC11054173; doi:10.3390/tomography10040041)
Supplement: Supplementary file 1 [file tomography-10-00041-s001.zip › tomography-2823156-supplementary.pdf]

Supplementary file 1. Changes in number of the US B-lines at baseline and at the 12 months of follow-up.

Patient number	Number of B-lines (baseline)	Number of B-lines (12 months)
1	15	19
2	13	25
3	2	11
4	14	22
5	2	11
6	1	24
7	3	12
8	2	11
9	0	8
10	15	24
11	5	11
12	2	8
13	15	26
14	5	21
15	2	11
16	2	9
7	15	21
18	5	15
19	2	10
20	30	34
21	2	12
22	2	9
23	13	24
24	14	24
25	2	13
26	5	18
27	2	8
28	15	28
29	3	13
30	4	13
Mean	7.1	16.5
95%CI for the mean	4.52 - 9.74	13.8 -19.19
SD	6.9	7.2
p	<0.0001	<0.0001

95%CI = 95% confidence interval; SD = standard deviation.

Supplementary file 2. Borg Dyspnoea scale and pulmonary US progression during 1-year of follow-up.

Borg Dyspnea Scale, n (%)		Pulmonary US score, n (%)		*p*
Basal	133	Basal	133	
0	111 (83.5)	0	54 (40.6)	0.001
0.5	22 (16.5)	1	51 (38.3)	
1	0	2	28 (21)	
2	0	3	0	
3	0	0	0	
3 months	130	3 months	130	
0	106 (81.4)	0	51 (39.2)	0.001
0.5	24 (18.6)	1	49 (37.7)	
1	0	2	30 (23.1)	
2	0	3	0	
3	0	0	0	
6 months	125	6 months	125	
0	97 (77.40)	0	45 (36.00)	0.001
0.5	25 (20.2)	1	49 (39.20)	
1	3 (2.4)	2	31 (24.80)	
2	0	3	0	
3	0	0	0	
9 months	123	9 months	123	
0	90 (73.2)	0	39 (31.71)	0.001
0.5	26 (21.1)	1	50 (40.65)	
1	6 (4.9)	2	34 (27.64)	
2	1 (0.8)	3	0	
3	0	0	0	
12 months	121	12 months	121	
0	86 (71.1)	0	29 (23.97)	0.001
0.5	24 (19.8)	1	53 (43.80)	
1	6 (5.0)	2	38 (31.40)	
2	4 (3.3)	3	1 (0.83)	
3	1 (0.8)	0	0	
